# Associations between Maternal Adverse Childhood Experiences, Stressful Life Events, and Child Behavioral Outcomes in Racially/Ethnically Diverse Families

**DOI:** 10.1007/s10903-025-01816-5

**Published:** 2025-11-22

**Authors:** Lisa Zak-Hunter, Christian Okitondo, Allan Tate, Kaitlyn Mulhern, Abby Brustad, Laura Miller, Jerica M. Berge

**Affiliations:** 1 Department of Family Medicine and Community Health, The University of Minnesota School of Medicine, Minneapolis, MN USA; 2 College of Public Health, Department of Epidemiology and Biostatistics, The University of Georgia, Athens, GA USA; 3 Department of Family Medicine and Adult and Child Center for Outcomes Research and Delivery Science, The University of Colorado Anschutz Medical Campus, Aurora, CO USA

**Keywords:** Adverse childhood experiences (ACEs), Stressful life events (SLEs), Maternal health, Racially/ethnically diverse women, Childhood behavior

## Abstract

The purpose of this study was to examine associations between psychosocial factors and parenting practices with behavioral outcomes in children from racially/ethnically diverse mothers with a history of adverse childhood experiences (ACEs) and stressful life events (SLEs). This was a secondary analysis of cross-sectional survey data from the *Family Matters* cohort study. The analytic sample included families with children ages 5–9 with non-White mothers (*n* = 930) recruited from Minneapolis-St. Paul primary care clinics representing five racial/ethnic backgrounds (Black, Native American, Hmong, Somali, Latino). Multivariable regression models examined associations between maternal ACEs/SLEs, parenting practices, and child behavioral outcomes, adjusting for sociodemographic and contextual factors. Women with *≥* 4 ACEs and/or SLEs reported more permissive (*p* = < 0.001) and authoritarian parenting (*p =.*0018), substance use (*p = <.*001), and levels of moderate and severe psychosocial distress (*p = <.*001) compared to those with fewer to no ACEs/SLEs. Child behaviors were elevated among mothers reporting severe psychosocial distress (β = 2.85; *p* < .001), permissive parenting (β = 1.88; *p* < .001), and authoritarian parenting (β = 1.27; *p* < .001). There was a significant interaction between authoritarian parenting and community violence (β = 0.714, *p* = .022), strengthening the association between maternal mental health and child behavioral difficulties. In racially/ethnically diverse women with a history of ACEs/SLEs, maternal mental health distress and parenting practices were independently associated with higher child behavioral problems. Community violence may also play a role in these relationships. It is important to maintain maternal wellbeing by connecting mothers with appropriate mental health and parenting resources.

**Supplementary Information:**

The online version contains supplementary material available at 10.1007/s10903-025-01816-5.

## Introduction

Exposure to adverse childhood experience (ACEs) and stressful life events (SLEs) negatively affect adult substance use, mental health, physical health, and relationships [1]. ACEs are stressful and sometimes traumatizing events that occur to persons under age 18. Examples include parental mental illness, abuse/neglect, or parental divorce. SLEs are stressful events that occur any time throughout the life course such as economic strain, job loss, or death of a family member [2, 3]. Toxic stress has been posited as a key underlying mechanism responsible for the physiological and psychological outcomes related to ACE/SLE exposure [4]. Toxic stress is the chronic activation of stress systems without sufficient buffers. It can affect executive functioning, problem solving, reward pathways, and lifespan stress response [5, 6]. Without appropriate bolstering of protective factors, effects can be transmitted intergenerationally [7–9].

Mothers with a history of ACEs/SLEs, may experience changes to cognitive functioning, mental health, and wellbeing which can influence their children’s health and wellbeing [10–22]. Different factors have been identified as impacting this relationship such as maternal substance use, mental health (e.g., depressive and anxious symptoms), parenting style (i.e., authoritative, authoritarian, permissive), and economic strain [14, 22–25]. However, there is less clarity to how these factors play a role in child internalizing (e.g., depressive symptoms, anxiety) and externalizing (e.g., hyperactivity, conduct problems) behavioral outcomes in racially/ethnically diverse populations, including those not born in the United States.

Racially/ethnically diverse groups and immigrants face a disproportionate number of ACEs [26] and social determinants of health (SDOH), both of which are associated with mental, physical, and behavioral health concerns [21, 22, 26]. Lack of access to education, healthcare, economic stability, neighborhood environment, and social, relational, and community context, are related to poor outcomes in pediatric mental and behavioral health [27]. Racial and ethnic minority children exposed to compounded community trauma (violence at home and in the community) report poorer mental health outcomes, including higher anxiety, Post-Traumatic Stress Disorder (PTSD), and externalizing behaviors [28–30]. Studies from our own research team have shown significant associations between higher parental exposure to ACEs and higher child body mass index (BMI) and lower child mental health (e.g., higher depressive symptoms and conduct problems) in racially/ethnically diverse households [31, 32].

Importantly, immigration experiences are uniquely stressful and sometimes traumatizing. While first generation immigrants often demonstrate lower mental health concerns than the general population, their experiences pre- and post-migration play a significant role in their long-term outcomes [33, 34]. Common stressors include adversity in their country of origin, stressors in the immigration process (e.g., harsh living conditions, exposure to violence), and challenges with resettlement (e.g., language barriers, loss of community/family support, unemployment, and adaptation to new social policies) [33]. Tensions in raising children in a foreign country affect parents and children alike. Children of immigrants display higher rates of depression, PTSD, and disruptive behaviors [35, 36]. They appear to struggle more than first generation groups due to tensions balancing social norms and cultural expectations [36]. Therefore, when examining the outcomes of children across diverse groups, it is important to take maternal birthplace and acculturation into consideration.

Overall, ACEs, SLEs and SDOH are intricately linked to maternal and child behavior outcomes in diverse groups. Maternal birthplace may also play a role as immigrant experiences are often interconnected with these outcomes. Additionally, stressful life events have not always been measured using the ACEs framework, which is important to examine to further understand how these factors interplay within their associations with child behavioral outcomes. Thus, the purpose of this paper is to examine factors that influence internalizing and externalizing behavioral outcomes in children of racially/ethnically diverse and immigrant women who have experienced ACEs and/or SLEs.

## Conceptual Framework

This study is guided simultaneously by Family Stress Theory [38] and the Ecological Systems Framework [39]. Family Stress Theory explains how critical stressful events (e.g., ACEs/SLEs) impact family members individually (e.g. maternal mental health, parenting, substance use), and how that shapes other family members’ lived experiences, which may be maladaptive (e.g., pediatric externalizing and internalizing behaviors). This is couched within multiple ecological layers that also impact child behavioral outcomes such as: individual (i.e., child race, sex), microsystem (i.e., mother variables (ACEs/SLEs, depression, parenting, substance use), family income, and economic strain), and macrosystem (i.e., neighborhood violence, social capital) to ultimately influence childhood behavioral outcomes. These two conceptual frameworks informed our research questions which include: (1) Are ACEs/SLEs correlated with maternal mental health, substance use, and parenting styles in racially/ethnically diverse women? (2) How are maternal behavioral factors and mental health related to child mental health? (3) How does maternal nativity status, community violence, and history of ACEs/SLEs modify the relationship between maternal health outcomes and children’s behavioral outcomes? Based on previous research, we hypothesized that maternal mental health, substance use, and parenting practices would be correlated with ACEs/SLEs and that, in turn, would be correlated with child internalizing and externalizing behaviors. We also hypothesized that maternal nativity, community violence, and higher exposure to ACEs/SLEs would be associated with poorer childhood behavioral outcomes.

## Methods

### Participants/Data Collection

This paper presents a secondary analysis of cross-sectional data derived from the *Family Matters* study [40–42], a longitudinal, mixed-methods observational cohort study designed to investigate childhood cardiovascular disparities and corresponding risk and protective factors. The study recruited participants from primary care clinics in Minneapolis-St. Paul, Minnesota. Eligibility criteria for parents/guardians included: having a child aged 5–9 years old, residing in the region, and proficiency reading in English, Spanish, Hmong, or Somali languages. The exclusion criteria encompassed children with dietary restrictions, body mass index below the 5th percentile, and those diagnosed with severe and persistent mental illness. To ensure diversity in the population and subpopulation analyses, the *Family Matters* study stratified recruitment of children from six racial/ethnic groups: White, Black, Native American, Hmong, Somali, and Latino, following approval by the University of Minnesota’s Institutional Review Board. This study’s sample included all nonwhite women (*n* = 930), including Black (*n* = 240), Native American (*n* = 197), Hmong (*n* = 195), Somali (*n* = 108), and Latino (*n* = 190). Mothers’ average age was 34 years (SD = 7.9) and children’s was 7 (SD = 1.5). In line with the ethical principles outlined in the Declaration of Helsinki, all parents provided informed consent before participating in the study.

### Measures

This study examined associations between maternal mental health, substance use, and parenting practices/style with child behavioral outcomes among racially/ethnically diverse women exposed to ACEs and SLEs. **Independent variables** included mental health, self-reported substance use, and parenting style. A comprehensive measures table is included in supplemental Table 1 [43–53]. The primary exposure variable was mothers’ report of ACEs (11 ‘yes/no’ items from the CDC-Kaiser ACE study [51]) and SLEs (13 ‘yes/no’ items from the List of Threatening Experiences [52]). Example ACE questions: “Did a household member go to prison?”, “Were your parents ever separated or divorced. Example SLE questions: “Have you been attacked, beaten, or mugged?”, “Have you had a major financial crisis?”. These were operationalized as a categorical variable (no ACEs/SLEs, 1–3 ACEs/SLEs, and *≥* 4 ACEs/SLEs). Maternal outcomes included self-reported mental health: stress levels, stress coping efficacy, and psychosocial distress (measured by the Kessler Psychological Distress Scale (K6)), substance use, and parenting style. The K6 assessed nervousness, hopelessness, and restlessness on a 5 point Likert scale; scores >5 were moderate or severe distress and scores *≥* 13 severe distress [44]. Substance use included cigarette, alcohol, and marijuana use on a 5-point Likert scale; recoded 0–4 to create a composite score. Parenting style was measured using a shortened version of the Parenting Practices Questionnaire [46], examining permissive, authoritarian, and authoritative styles on a 5-point Likert scale. Authoritative style focused on communication, rule explanation, and self-expression (e.g., “I encourage my children to express themselves”), authoritarian emphasized physical discipline, yelling, and obedience (e.g., “When my child asks why they must obey, I say: Because I said so”), and permissive demonstrated inconsistent discipline, yielding to demands, and discipline difficulty (e.g., “I give in to my child when they cause a commotion”). Mean scores were computed per style [54]. The PPQ has demonstrated strong construct validity and internal reliability (α ≈ 0.75–0.88) across culturally diverse samples [46]. For **dependent variables**, child behavioral outcomes were measured using the Strengths and Difficulties Questionnaire (SDQ) [49]. The SDQ has four subdomains: emotional symptoms, conduct problems, hyperactivity/inattention, and peer relationship problems. Parents rated items as “not true”, “somewhat true”, or “certainly true”. A total score (0–40) was computed by summing the subscales. Higher scores reflect greater difficulties. Sociodemographic characteristics included child age, mother age, mother nativity status, education, relationship status, income sufficiency, and household income.

### Statistical Analysis

Descriptive statistics were computed, and bivariate tabular, statistical tests (two-tailed, type I error 0.05) were conducted to examine the correlation between ACEs/SLEs and sociodemographics. Multiple regression for continuous outcomes (child total difficulty score, parenting practices, frequency of substance use, and psychosocial distress and anxiety scales) and logistic regression for binary outcomes (caregiver moderate and severe psychosocial distress and anxiety indicators) were used to assess relationships between ACEs/SLEs and parent and child mental health and parenting behaviors. All regression models were multivariate. When exploring the associations between ACEs/SLEs and parent outcomes, the model controlled for basic demographic variables: child sex, age, maternal age, race/ethnicity, and maternal nativity. In contrast, when assessing the impact of mother outcomes on child mental health, additional controls such as family functioning, community violence, social capital, and acculturation were added to capture the complex environmental and socio-cultural influences which can disproportionately impact minority groups. Interactions of all socio-environmental factors, parenting practices, and maternal mental health on the relationship between maternal adverse life experiences and child difficulties were examined. There was no statistical evidence (at *p* <.05) that the ACEs/SLEs-child difficulties relationship depended on these other factors, except for authoritarian parenting. Main effects results were retained for non-significant interactions. Robust standard errors, specifically Huber sandwich estimator, were computed to address potential model misspecification, and all analysis and data management was conducted using Stata 17.0 MP software (College Station, TX).

## Results

The demographic characteristics are presented in Table 1. Three quarters of mothers endorsed at least one ACE or SLE and almost one third reported *≥* 4 ACEs/SLEs. Those who endorsed greater exposure to ACEs/SLEs were more likely to be non-immigrant, single caregivers, with annual household incomes less than $20,000 that were difficult to live on. The racial/ethnic composition of the “Parent born in the U.S.” group was diverse, with the largest proportions being Black/African American (42.2%) and Native American (35.0%). Detailed distributions of parent nativity status by race/ethnicity are available in Supplemental Table 2.

**Table 1:** Demographic characteristics of Racially and ethnically diverse women in the *Family matters* study (*n* = 930 mothers)

	Racially and ethnically diverse women who reported no ACE or SLE (*n* = 216)	Racially and ethnically diverse women who reported 1–3 ACE or SLE (*n* = 426)	Racially and ethnically diverse women who reported at least 4 ACE or SLE (*n* = 288)	Overall *P*-value
**Child Age in years (SD)**	7.18 (1.52)	7.23 (1.52)	7.00 (1.49)	0.1224
**Parent Age in years (SD)**	34.52 (7.62)	34.89 (8.52)	33.66 (7.17)	0.1272
**Parent Born in the U.S.**	69 (31.9%)	263 (61.7%)	216 (75.0%)	**< 0.001**
**Parent Educational Attainment**				0.044
Some high school	47 (21.8%)	60 (14.1%)	49(17.0%)	
High school or Associates	108 (50.0%)	195 (45.8%)	136(47.2%)	
Some College or Bachelors	44 (20.4%)	133 (31.2%)	84(29.2%)	
Graduate Degree	17 (7.9%)	38 (8.9%)	19 (6.6%)	
**Relationship Status**				**< 0.001**
Married	106 (49.1%)	139 (32.6%)	67 (23.3%)	
Committed relationship or engaged	27 (12.5%)	82 (19.3%)	75 (26.0%)	
Separated/Divorced	19 (8.8%)	44 (10.3%)	24 (8.3%)	
Widowed	5 (2.31%)	3 (0.7%)	3 (1.0%)	
Single	59 (27.3%)	158 (37.1%)	119 (41.3%)	
**Depression**				**< 0.001**
No	214 (99.1%)	376 (88.3%)	178 (61.8%)	
Yes	2 (0.9%)	50 (11.7%)	110 (38.2%)	
**Live on Income**				**< 0.001**
Not at all difficult	101 (46.8%)	109 (25.6%)	32 (11.1%)	
Somewhat difficult	102 (47.2%)	233 (54.7%)	135 (46.9%)	
Very difficult or can barely get by	10 (4.6%)	61 (14.3%)	89 (30.9%)	
Extremely difficult or impossible	3 (1.4%)	23 (5.4%)	32 (11.1%)	
**Household Income**				**< 0.001**
Less than $20,000	61 (28.2%)	145 (34.0%)	134 (46.5%)	
$20,000 - $34,999	77 (35.6%)	122 (28.6%)	74 (25.7%)	
$35,000 - $49,999	42 (19.4%)	82 (19.2%)	42 (14.6%)	
$50,000 - $74,999	19 (8.8%)	48 (11.3%)	29 (10.1%)	
$75,000 - $99,999	5 (2.3%)	15 (3.5%)	6 (2.1%)	
$100,000 or more	8 (3.7%)	12 (2.8%)	1 (0.3%)	
Not reported	4 (1.9%)	2 (0.5%)	2 (0.7%)	

### RQ 1. Are ACEs/SLEs Correlated with Maternal Mental health, Substance use, and Parenting Style among Racially and Ethnically Diverse women?

The adjusted associations between ACEs/SLEs and substance use behaviors and parenting styles were examined. ACEs/SLEs were overall correlated with higher self-reported substance use (*p* <.0001) and permissive (*p* <.0001) and authoritarian parenting (*p* =.0018). There was a nonlinear pattern of association between substance use and permissive parenting suggesting that at the *≥* 4 ACEs/SLEs threshold, these behaviors were more common compared to no ACEs/SLEs and the middle category (1–3 ACEs/SLEs). Additionally, ACEs/SLEs were approximately linearly related across the three ACEs/SLEs categories for authoritarian parenting. The strongest ACEs/SLEs effect against the no ACEs/SLEs reference category was observed for permissive parenting (mean difference: 0.410; 95% CI: 0.264, 0.555).

The ACEs/SLEs-mental health relationships examined binary indicators of psychological distress and anxiety. There was strong statistical evidence that ACEs/SLEs were correlated with severe and moderate-severe distress and anxiety in mothers (*p* <.001). About 1 in 3 more mothers reported moderate or severe distress (probability difference (PD): 0.377; 95% CI: 0.296, 0.457) and about 1 in 5 more mothers reported moderate or severe anxiety (PD: 0.192; 95% CI: 0.126, 0.259) if they had *≥* 4 ACEs/SLEs compared to none. The magnitude of only severe distress and anxiety associations indicated a threshold effect increase at the *≥* 4 ACEs/SLEs category compared to the association observed at the middle ACEs/SLEs category. The strongest threshold effect size was observed for the *≥* 4 ACEs/SLEs Distress-PD (0.168) compared with the 1–3 ACEs/SLEs Distress-PD (0.041) which suggested there were about 12 per 100 more mothers living with prevalent, severe psychological distress among the highest ACEs/SLEs category compared with the middle ACEs/SLEs category (see Table 2).

**Table 2:** Adjusted relationships between adverse life experiences (ACEs) and stressful life events (SLEs) with maternal substance Use, parenting Style, and mental health (*n* = 930 Racially/Ethnically diverse Mothers)

Part A. Adjusted Linear Regression				
Predictor	Outcomes			
	Substance Use			
Numbers of ACEs/SLEs (Ref: No ACE/SLE)	**β Coefficient**	**L 95% CI**	**H 95% CI**	**Overall** **P-value**
1–3 ACEs/SLEs	0.078	−0.006	0.163	**< 0.001**
At least 4 ACEs/SLEs	0.310	0.201	0.420	
	Permissive Parenting			
1–3 ACEs/SLEs	0.152	0.029	0.275	**< 0.001**
At least 4 ACEs/SLEs	0.410	0.264	0.555	
	Authoritative Parenting			
1–3 ACEs/SLEs	0.045	−0.106	0.196	0.8340
At least 4 ACEs/SLEs	0.026	−0.133	0.185	
	Authoritarian Parenting			
1–3 ACEs/SLEs	0.119	0.002	0.237	**0.0018**
At least 4 ACEs/SLEs	0.239	0.106	0.371	
Part B. Adjusted Logistics Regression				
	Outcomes			
	Severe Parental Distress			
Numbers of ACEs/SLEs (Ref: No ACE/SLE)	**Predicted Probability Difference**	**L 95% CI**	**H 95% CI**	**Overall** **P-value**
1–3 ACEs/SLEs	0.041	0.009	0.073	**< 0.001**
At least 4 ACEs/SLEs	0.168	0.115	0.220	
	Moderate or Severe Parental Distress			
1–3 ACEs/SLEs	0.110	0.043	0.177	**< 0.001**
At least 4 ACEs/SLEs	0.377	0.296	0.457	
	Severe Parental Anxiety			
1–3 ACEs/SLEs	0.015	−0.014	0.044	**0.0002**
At least 4 ACEs/SLEs	0.090	0.044	0.136	
	Moderate or Severe Parental Anxiety			
1–3 ACEs/SLEs	0.047	−0.002	0.096	**< 0.001**
At least 4 ACEs/SLEs	0.192	0.126	0.259	

*Models controlled for Child gender, child age, parent age, parent race & ethnicity, and parent nativity. Statistical significance value set to 0.05 level*±

Sample Interpretation for Linear Regression: Compared to mothers with no history of ACE/SLE, those with 1-3 ACEs/SLEs had a small increase in their substance use score (β coefficient = 0.078), and mothers with at least 4 ACEs/SLEs had a substantially higher substance use score (β coefficient = 0.310) compared to those with no ACEs/SLEs. These differences are statistically significant (p-value < .0001)±

Sample Interpretation for Logistic Regression: Compared to mothers with no ACEs/SLEs, those with 1-3 ACEs/SLEs have a 4.1% higher probability of experiencing severe parental distress (predicted probability difference = 0.041), and those with at least 4 ACEs/SLEs have a 16.8% higher probability of experiencing severe parental distress (predicted probability difference = 0.168). The overall p-value is statistically significant (p-value < .0001)

### RQ 2. How Are Maternal Behavioral Factors and Mental Health Related To Child Mental Health?

Maternal substance use, parenting style, and mental health were examined as correlates of child mental health. Maternal substance use (*p* =.515) and authoritative parenting (i.e., high responsiveness and high expectations) (*p* =.846) were not associated with child internalizing and externalizing behavioral difficulties. Permissive parenting (i.e., low responsiveness and low expectations) was the strongest behavioral correlate of child internalizing and externalizing behaviors (β: 1.88; 95% CI: 1.39, 2.36; *p* <.001), followed by authoritarian parenting (i.e., low responsiveness and high expectations/control) (β: 1.27; 95% CI: 0.67, 1.87; *p* <.001). Maternal mental health was the strongest overall predictor of child internalizing and externalizing behaviors (*p <.001*). Severe mental health distress followed by moderate-severe anxiety was most strongly related to child internalizing and externalizing concerns (β: 2.85; 95% CI: 1.58, 4.12 and β: 2.83; 95% CI: 1.86, 3.79, respectively) (see Table 3).

**Table 3:** Adjusted associations between parent substance Use, parenting Style, and mental health on child internalizing and externalizing behavior score (*n* = 930 parent-Child Dyads)

Adjusted Linear Regression results				
	Total Difficulty Score			
**Predictors**	**β**	**Low 95% CI**	**High 95% CI**	**P-Value**
Substance Use	0.19	−0.38	0.76	0.515
Permissive Parental	1.88	1.39	2.36	**< 0.001**
Authoritative Parental	0.05	−0.41	0.51	0.846
Authoritarian Parental	1.27	0.67	1.87	**< 0.001**
Severe Parental Distress	2.85	1.58	4.12	**< 0.001**
Moderate or Severe Parental Distress	1.97	1.20	2.74	**< 0.001**
Severe Parental Anxiety	2.68	1.11	4.24	**< 0.001**
Moderate or Severe Parental Anxiety	2.83	1.86	3.79	**< 0.001**

*Models adjusted for: child gender, child age, parent age, parent race & ethnicity, parent nativity status, live on income, family malfunction, violence, social capital, and acculturation

± Sample Interpretation:

For the severe parental distress row, the β coefficient of 2.85 suggests an increase of almost 3 points in children’s Total Difficulty Score with every one unit increase in the mother’s severe distress

### RQ 3. How Does Maternal Nativity status, Community violence, and History of ACEs/SLEs Modify the Relationship between Maternal Health Outcomes and Children’s Total Difficulty score?

We explored the potential moderating role of maternal nativity status, community violence, and history of ACEs/SLEs on the association between maternal health outcomes and child behavioral outcomes. The results revealed that community violence significantly modified the influence of authoritarian parenting on children’s behaviors (β = 0.714, 95% CI: 0.103, 1.325, *p* =.022), suggesting the negative effect of poor maternal health on children’s behaviors is stronger when mothers experience high levels of community violence and exhibit higher levels of authoritarian parenting. Maternal nativity status and history of ACEs/SLEs did not moderate the relationship between maternal health outcomes and children’s behaviors.

## Discussion

This study examined how maternal mental health and parenting factors, in the setting of maternal exposure to ACEs/SLEs, related to childhood internalizing and externalizing behaviors in racially/ethnically diverse groups. We examined how other socio-cultural factors like family functioning, neighborhood violence, and maternal nativity status influenced this relationship. Our sample resembled those in larger studies with regard maternal age and ACEs/SLEs exposure [8, 55]– [56], supporting the idea that younger, racial/ethnically diverse women are at greater risk of experiencing ACEs/SLE [57].

Our findings both support and extend existing research. First, a history of ACEs/SLEs was related to maternal substance use, less healthy parenting style (authoritarian, permissive), and elevated psychological distress. Mothers with *≥* 4 ACEs/SLEs, had higher substance use, permissive parenting, and about 12% of these mothers experienced severe psychological distress. This is consistent with research linking ACEs/SLEs to maternal mental health, substance use, and parenting practices [2, 8, 24, 51, 56, 58–61]. Our findings extend prior research by showing that these patterns occur in diverse and immigrant mothers too.

Severe mental health distress and less healthy parenting were related to child behavioral outcomes, with mental health having a stronger relationship. This supports research that maternal depression and anxiety impact the relationship between maternal ACEs/SLEs and child behavior outcomes [24, 60–63]. Likewise, permissive and authoritarian parenting styles also were related to internalizing and externalizing symptoms in children. Previous literature that has demonstrated mothers with ACEs/SLEs utilize negative or hostile parenting practices [25, 60, 61]. ACEs may be linked with maternal psychopathology and emotional regulation, which interferes with parenting practices, and increases the risk of child behavior concerns [60]. Our findings seem to support this. It is also possible these mothers are more attuned to their children’s behavior, which may influence their report of these concerns and parenting approaches. These findings support and emphasize the need for targeting maternal mental health and parenting in diverse populations through public health and clinical interventions.

Using a theoretical framework, we examined whether social and contextual variables (e.g., community violence, parental nativity and parenting style, social capital, maternal ACEs/SLEs) impact the relationship between maternal mental health and child behavior outcomes [25, 27, 29, 30, 37]. We found two variables that interact: community violence authoritarian parenting style. Specifically, maternal psychological distress was a stronger risk factor for child outcomes in violent neighborhoods and when authoritarian parenting was used. This supports research demonstrating that parents living in violent communities tend to utilize more harsh parenting to keep their children safe but this can also adversely impact their mental health [64]. The interaction between maternal mental health, community violence, authoritarian parenting style, and child behaviors is a new finding and has implications for research and clinical practice. Specifically, public health interventions aiming to mitigate the effects of ACEs and SLEs in racially/ethnically diverse groups should consider addressing mental health distress in mothers and the impact of community violence and parenting style to reduce child behavioral difficulties.

Strengths of this study are its large size and racial/ethnic diversity. However, there are limitations. Findings from the five specific groups may not be generalizable to other low-income minority populations. Also, the ACE/SLE measures may not capture the experiences of non-US-born mothers, leading to potential underreporting of trauma specific to their cultural group or experience. Because participants were recruited from primary care clinics, this sample also may not accurately reflect the experiences of those with limited access to health care. Additionally, all measures were self-reported and part of a larger observational cohort study [31, 40]. Answers could have been affected by factors such as social desirability or a want for greater intervention or resources.

## New Contributions To the Literature

This study informs clinical and public health interventions both for mothers and the community to support racially/ethnically diverse families exposed to ACEs/SLEs. Addressing maternal mental health is crucial for reducing child behavioral concerns. Interventions may include integrated behavioral health care response to regular depression screening or group therapy tailored to racial/ethnic minority mothers in primary care. Secondly, partnering with cultural community agencies to teach and reinforce positive parenting practices may be helpful. Lastly, addressing the impact of community violence in mothers with ACEs/SLEs could have significant positive impact on the wellbeing of children from racially/ethnically diverse groups.

## Supplementary Information

Below is the link to the electronic supplementary material.

Supplementary Material 1

## Data Availability

Deidentified individual participant data will not be made available.
