# Supplementary material for: Associations between Maternal Adverse Childhood Experiences, Stressful Life Events, and Child Behavioral Outcomes in Racially/Ethnically Diverse Families
Source: J Immigr Minor Health. 2025 Nov 22;28(3):637–46. doi: 10.1007/s10903-025-01816-5 (PMC13222239; doi:10.1007/s10903-025-01816-5)
Supplement: Supplementary file 1 — Supplementary Material 1 [file 10903_2025_1816_MOESM1_ESM.docx]

**Supplemental Table 1 - Predictor and Outcome Variables Description and Operationalization Methods**

| **Variables** | | **Racially and ethnically diverse women who reported no ACE or SLE**  **(n = 216)** | **Racially and ethnically diverse women who reported 1 -3 ACE or SLE**  **(n = 426)** | **Racially and ethnically diverse women who reported at least 4 ACE or SLE**  **(n = 288)** |
| --- | --- | --- | --- | --- |
| **Adverse Childhood Experiences (ACEs) and Stressful Life Events (SLEs)** | | | | |
| ACEs | Participants were surveyed to report their ACES, which involved answering a series of the following yes or no questions: Were your parents ever separated or divorced? Was a household member depressed or mentally ill, or did a household member attempt suicide? Did a household member go to prison (including yourself)? Did you often or very often feel that you didn't have enough to eat, had to wear dirty clothes, and had no one to protect you? Did you often or very often feel that your family didn't look out for each other, feel close to each other, or support each other? Did you often or very often feel that your parents were too drunk or high to take care of you? Was your mother or stepmother often or very often pushed, grabbed, slapped, or had something thrown at her? Did a parent or other adult in the household often or very often swear at you, insult you, put you down, or humiliate you? Were you physically abused by a parent or guardian? Were you sexually abused by a parent or guardian? Were you sexually abused by someone who was not a parent or guardian?^47^ The number of yes responses were totaled and multiplied by 10 so in this analysis the associations are in 10% units.^47^ | 0 (0) | 1.17 (0.97)  Almost 48% responded reported that their parents were separated or divorced | 4.76 (2.81)  Almost 75% responded reported that their parents were separated or divorced |
| SLEs | SLEs were reported via survey and participants answered "No", "Yes, in the past year", or "Yes, more than a year ago" to the following questions: Had a serious illness Were fired from your job Had a major financial crisis Had problems with the police or a court appearance Had a separation due to marital difficulties or broke off a steady relationship Had a parent, child, spouse/significant other, close relative or close friend die Been in a serious car accident or another kind of accident at work or somewhere else Been involved in a dating or marital relationship where you felt controlled, intimidated, or threatened Been hit, shoved, held down or had some other physical force used against you by a spouse/significant other or someone you were dating Been attacked, beaten, or mugged Had a close family member or friend die violently, for example in a serious car crash, mugging, homicide, or suicide Witnessed a situation in which someone was seriously injured or killed, or in which you feared someone would be seriously injured or killed?^48^ For this analysis, the number of yes responses were totaled and multiplied by 10 so in this analysis the associations are in 10% units.^47^ | 0 (0) | 1.16 (1.0)  About 28% of respondents reported that they had a parent, child, spouse/significant other, close relative or close friend die. | 3.83 (2.96)  About 67% of respondents reported that they had a major financial crisis |
| **Maternal Behavioral and Mental Health** | | | | |
| Substance Use | Substance use was reported via survey on a five-point Likert scale: "1, Never", "2, A few times", "3, Monthly", "4, Weekly", "5, Daily." Participants provided a response across three domains: cigarettes, beer/wine/hard liquor, and marijuana. This scale was recoded zero to four where never was coded as zero. The mean across the three domains was calculated for each participant.^39^ | 0.24 (0.50) | 0.50 (0.63) | 0.81 (0.80) |
| Parental Distress | Mental health distress was measured via a survey, utilizing a five-point Likert scale with options ranging from "1, All of the time" to "5, None of the time." Participants' responses were collected across six domains: nervousness, hopelessness, restlessness or fidgetiness, depression, a feeling that everything was an effort, and worthlessness. These variables were reverse scored for analysis. The assessment of mental health distress was then dichotomized using the K6 scale. Participants with a K6 score higher than 13 were classified as experiencing severe mental health distress, while those with a score above 5 were considered to have moderate or severe distress.^40^ | 2.38 (3.96)  6.5% reported severe distress and 15.7% reported moderate or severe distress | 4.05 (4.68)  6.9% reported severe distress and 28.6% reported moderate or severe distress | 7.40 (6.29)  19.1% reported severe distress and 54.2% reported moderate or severe distress |
| Anxiety | Anxiety was reported via survey and participants answered based on a five-point Likert scale: "1, All of the time", "2, Most of the time", "3, Some of the time", "4, A little of the time", "5, None of the time." Participants provided responses across four domains: not being able to stop or control worrying, trouble relaxing, becoming easily annoyed or irritable, and feeling afraid as if something awful might happen. The variables representing the four domains were reverse scored. Anxiety was coded as a dichotomous variable using the GAD7 scale. Participants with a score of nine or higher were coded as having severe anxiety. Participants with a score greater than six were classified as having moderate or severe anxiety.^41^ | 2.13 (2.42)  2.3% reported severe anxiety and 6.5% reported moderate or severe anxiety | 3.03 (2.58)  4.2% reported severe anxiety and 12.4% reported moderate or severe anxiety | 4.67 (3.05)  12.9% reported severe anxiety and 29.2% reported moderate or severe anxiety |
| **Maternal Parenting Style** | | | | |
| Permissive  (considered an unhealthy parenting style) | Permissive parenting style was reported via survey on a five-point Likert scale based on the Parenting Practices Questionnaire: "1, Never", "2, Once in a while", "3, About half of the time", "4, Very often", "5, Always." Participants provided responses across three domains: I threaten [child_name] with punishment more often than actually giving it, I give into [child_name] when he/she causes a commotion, I find it difficult to discipline [child_name].^42^ The responses were summed for each participant. The mean permissive parenting style score was then computed. | 1.55 (0.72) | 1.78 (0.75) | 2.09 (0.85) |
| Authoritative (considered the most healthy parenting style) | Authoritative parenting style was reported via survey on a five-point Likert scale based on the Parenting Practices Questionnaire: "1, Never", "2, Once in a while", "3, About half of the time", "4, Very often", "5, Always." Participants provided responses across three domains: I encourage [child_name] to talk about his/her problems, I give [child_name] reasons why rules should be obeyed, I encourage [child_name] to freely express him/herself even when disagreeing with me. The responses were summed for each participant. The mean authoritative parenting style score was then computed.^42^ | 4.20 (0.99) | 4.28 (0.83) | 4.33 (0.72) |
| Authoritarian  (considered the most unhealthy parenting style) | Authoritarian parenting style was reported via survey on a five-point Likert scale based on the Parenting Practices Questionnaire: "1, Never", "2, Once in a while", "3, About half of the time", "4, Very often", "5, Always." Participants provided responses across three domains: I use physical punishment as a way of disciplining [child_name] (e.g., spank, slap, grab) I yell or shout when [child_name] misbehaves When [child_name] asks why he/she has to obey, I state: because I said so, or I am your parent and I want you to. The responses were summed for each participant. The mean authoritarian parenting style score was then computed.^42^ | 1.82 (0.69) | 1.96 (0.68) | 2.08 (0.68) |
| **Family and Community Factors** | | | | |
| Family functioning | Family functioning as reported via survey on a four-point Likert scale: "1, Strongly disagree", "2, Somewhat disagree", "3, Somewhat agree", "4, Strongly agree." Participants provided responses across six domains: Family members are accepted for who they are, We avoid discussing our fears and concerns, We don't get along well together, We can express feelings to each other, In times of crisis we can turn to each other for support, We are able to make decisions about how to solve problems.^43^ | 2.89 (0.45) | 2.91 (0.37) | 2.85 (0.38) |
| Community violence | Neighborhood violence as reported via survey on a four-point Likert scale: "1, Never", "2, Once or twice", "3, A few times", "4, Many times." Participants provided responses across four domains:  Been awakened to the noise of police, ambulance, or gunshots, I have seen someone get arrested, I have seen drug deals, and I have seen someone beaten, stabbed or shot.^44^ | 1.58 (0.75) | 1.67 (0.80) | 2.17 (1.01) |
| Social capital | Social capital as reported via survey on a four-point Likert scale: "1, Strongly disagree", "2, Somewhat disagree", "3, Somewhat agree", "4, Strongly agree." Participants provided responses across three domains: People in my community/neighborhood help each other out, We watch out for each other's children in this community/neighborhood, and If children were outside playing and got hurt or scared, there are adults nearby who I would trust to help children.^45^ | 2.76 (0.87) | 2.65 (0.83) | 2.56 (0.79) |
| **Child Internalizing and Externalizing Behaviors** | | | | |
| SDQ | The SDQ, a 25-item open-access tool, screens youth behavioral problems based on the last 6 months. It includes 5 prosocial and 20 difficulty items, divided into scales for emotional symptoms, conduct problems, hyperactivity/inattention, peer relationship issues, and prosocial behaviors. Responses are on a "not true" to "certainly true" scale. The total difficulty score, excluding prosocial behavior, categorizes subjects as normal, borderline, or abnormal using cutoff points.^49^ | 11.25 (4.38) | 12.63 (4.40) | 14.49 (4.82) |
